# Olanzapine Use in Children and Adolescents With Anorexia Nervosa: A Narrative Synthesis of Efficacy, Safety and Applicability to UK Practice

**DOI:** 10.1192/bjo.2026.11942

**Published:** 2026-06-30

**Authors:** Sophie Nocton, Nicola Dawson, Charles Stanley

**Affiliations:** 1 Bradford District Care NHS Foundation Trust, Bradford, United Kingdom; 2 Leeds Community Healthcare NHS Foundation Trust, Leeds, United Kingdom

## Abstract

**Aims:**

Anorexia nervosa (AN) is a severe psychiatric disorder with the highest mortality of any mental illness. While psychological therapies such as family therapy remain first-line, up to 30% of adolescents experience partial or non-recovery, prompting interest in adjunctive pharmacological options. Olanzapine, an atypical antipsychotic associated with reduced cognitive rigidity and anxiety and with restorative weight gain, is increasingly used off-label in adolescent AN despite limited evidence and lack of NICE endorsement. This review aimed to synthesise international evidence on olanzapine use in under-18s with AN, evaluating efficacy, safety, tolerability and applicability to UK NHS practice.

**Methods:**

A systematic literature search was conducted across multiple bibliographic databases to identify peer-reviewed studies examining olanzapine use in patients under 18 years with anorexia nervosa for the period 2000 – 2025. Eligible study designs included randomised controlled trials (RCTs), open-label and observational studies, case series, audits and clinical guidelines. Findings were synthesised narratively to assess clinical outcomes, adverse effects and implementation within UK Child and Adolescent Mental Health Services (CAMHS).

**Results:**

Thirty-six studies met inclusion criteria. Evidence from adolescent RCTs was limited and underpowered, demonstrating modest effects on weight restoration and variable psychological benefit. In contrast, open-label and naturalistic studies consistently reported short-term improvements in body mass index and reductions in anxiety, rigidity and pre-meal distress, particularly at low doses (2.5 - 5mg/day). Sedation, increased appetite and mild metabolic changes were commonly reported and were generally reversible. A single case of neuroleptic malignant syndrome was identified. Engagement of families in shared decision-making was associated with improved adherence. UK prescribing data indicate ongoing off-label use within specialist CAMHS, reflecting a gap between clinical practice and formal guidance.

**Conclusion:**

Available evidence supports cautious, short-term, low-dose olanzapine use as an adjunct to multidisciplinary care for selected adolescents with treatment-resistant AN, with benefits appearing greatest for psychological symptoms rather than sustained weight restoration. However, the evidence base remains limited by small sample sizes, heterogeneous outcome measures and short follow-up periods. Within UK NHS CAMHS, safe prescribing requires consultant oversight, family consent and metabolic monitoring in line with national standards. Further adequately powered UK-based RCTs are required to clarify efficacy, acceptability and long-term outcomes.

